# Supplementary material for: Venomix: a simple bioinformatic pipeline for identifying and characterizing toxin gene candidates from transcriptomic data
Source: PeerJ. 2018 Jul 31;6:e5361. doi: 10.7717/peerj.5361 (PMC6074769; doi:10.7717/peerj.5361)
Supplement: Supplemental Information 3 [file peerj-06-5361-s003.gz › FinalOutput_E-6/Alpha-latroinsectotoxin-Lh1a_1/finaltree.pdf]

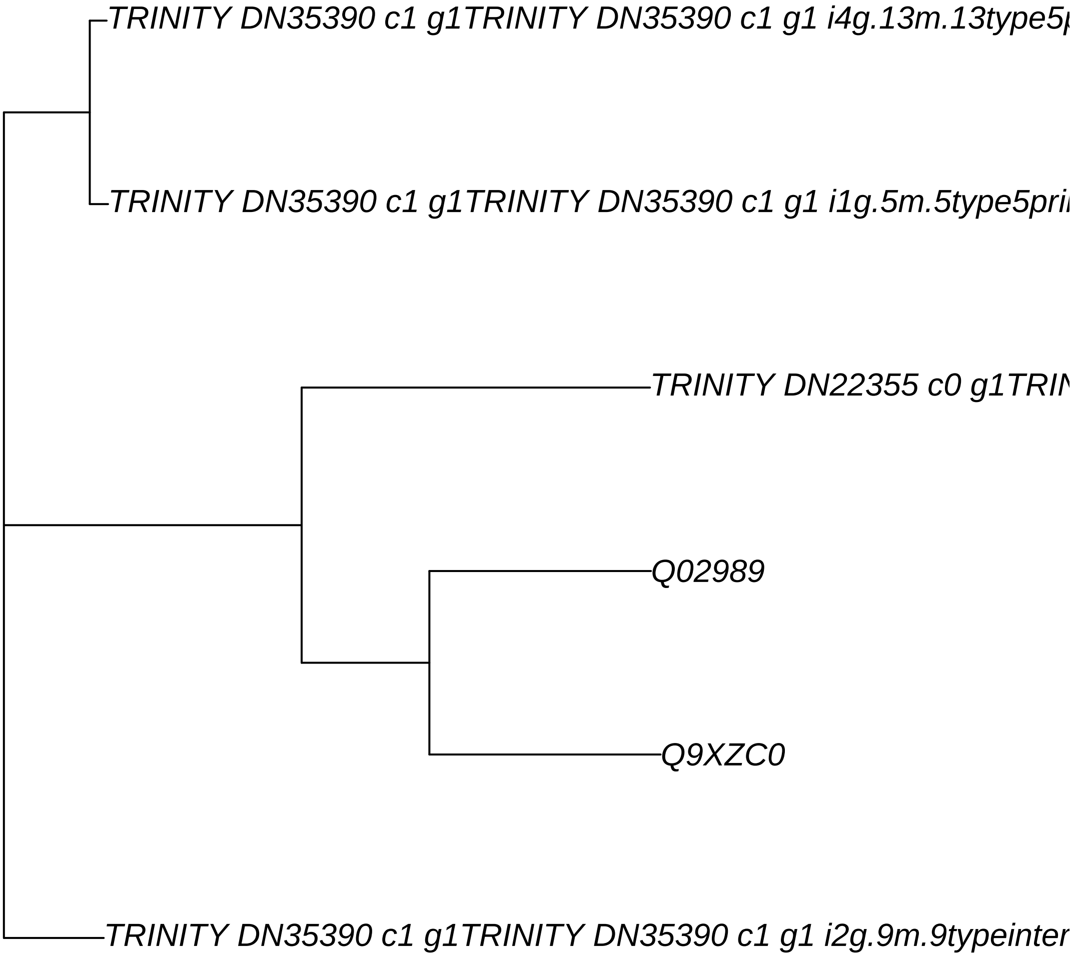
TRINITY DN35390 c1 g1TRINITY DN35390 c1 g1 i4g.13m.13type5
TRINITY DN35390 c1 g1TRINITY DN35390 c1 g1 i1g.5m.5type5pri
TRINITY DN22355 c0 g1TRIN
Q02989
Q9XZC0
TRINITY DN35390 c1 g1TRINITY DN35390 c1 g1 i2g.9m.9typeinter
